# Pan PPAR agonist stimulation of induced MSCs produces extracellular vesicles with enhanced renoprotective effect for acute kidney injury

**DOI:** 10.1186/s13287-023-03577-0

**Published:** 2024-01-02

**Authors:** Hongduk Kim, Seul Ki Lee, Sungok Hong, Tae Sub Park, Jimin Kim, Soo Kim, Tae Min Kim

**Affiliations:** 1 Institutes of Green-Bio Science and Technology, Seoul National University, Pyeongchang, Gangwon-do 25354 South Korea; 2 Brexogen Research Center, Brexogen Inc., Songpa-gu, Seoul, 05855 South Korea; 3 Graduate School of International Agricultural Technology, Seoul National University, Pyeongchang Daero 1447, Pyeongchang, Gangwon-do 25354 South Korea

**Keywords:** Acute kidney injury, Induced mesenchymal stem cells, Priming, Extracellular vesicle

## Abstract

**Background:**

Acute kidney injury (AKI) has a complex pathophysiology and imposes serious health concerns worldwide. Extracellular vesicles (EVs) derived from induced mesenchymal stem cells (iMSCs) have been recognized as novel cell-free therapeutics for various inflammatory and degenerative disorders. In this study, we investigated whether iMSCs stimulated with a pan-peroxisome proliferator-activated receptor (PPAR) agonist could enhance the therapeutic efficacy of EVs against AKI.

**Methods:**

Human iMSCs were primed with or without lanifibranor, a PPAR agonist for 24 h, and EVs were collected after an additional 24 h. The basic characteristics of EVs were evaluated using cryo-transmission electron microscopy imaging, immunoblot detection of EV markers, nanoparticle tracking analysis, and localization in AKI kidneys. In vitro, the potential of the EVs to promote the growth and survival of HK-2 cells undergoing cisplatin-induced apoptosis and anti-inflammatory effects in M1-polarized THP-1 was compared. Subsequently, AKI was induced in BALB/c mice using cisplatin. After 8 and 24 h of cisplatin treatment, iMSC-EVs or pan-PPAR-iMSC-EVs were injected intravascularly. At 96 h after cisplatin administration, the renoprotective effects of iMSC-EVs or pan-PPAR-iMSC-EVs in inhibiting inflammation and apoptosis were compared using serum biochemistry, histology, immunohistochemistry, and gene expression analysis by qPCR.

**Results:**

Both EV types expressed EV markers and had typical EV morphology, and their localization in the renal tissue was confirmed. The proliferation and survival of HK-2 cells were higher in pan-PPAR-iMSC-EVs than those in iMSC-EVs. In M1-polarized THP-1 cells, the reduction in the mRNA expression of inflammatory cytokines was more significant in pan-PPAR-iMSC-EVs than that in iMSC-EVs. In the mouse model of cisplatin-induced AKI, pan-PPAR-iMSC-EVs markedly enhanced renoprotective effects compared to iMSC-EVs. Specifically, pan-PPAR-iMSC-EVs reduced tissue inflammation, immune cell infiltration, and apoptosis. Pan-PPAR-iMSC-EVs also increased renal capillary density.

**Conclusion:**

Priming iMSCs with a PPAR agonist significantly improved the therapeutic potential of EVs by reducing inflammation and apoptosis. The reported strategy may contribute to the development of a novel cell-free option for AKI treatment.

*Trial registration:* Not applicable.

**Supplementary Information:**

The online version contains supplementary material available at 10.1186/s13287-023-03577-0.

## Background

Acute kidney injury (AKI) is a pathological condition in which a sudden loss of renal function occurs within hours to several days [1]. The increased incidence of AKI imposes a high healthcare burden and increased risk of chronic kidney disease (CKD) worldwide. It affects approximately 25% of hospitalized patients, and the risk of AKI in critically ill patients has been reported to be as high as 30 to 60% [2, 3]. AKI, with a complex pathophysiology, generally occurs during major surgery and is primarily caused by nephrotoxic drugs, ischemia, and contrast media [4]. In addition, risk factors, including age, hypertension, diabetes, and chronic renal disease, increase the mortality of patients with AKI [4]. Although several clinical trials have investigated the efficacies of various drugs, including diuretics (mannitol), antioxidants, and DPP4 inhibitors, the results have not been successful [5]. Therefore, innovative therapeutic strategies against AKI are needed [6]. 

Mesenchymal stem cells (MSCs) have been recognized as promising therapeutic tools for various diseases because they facilitate the recovery of damaged tissue and regulate immune tissue regeneration and responses [7]. Despite meaningful progress, the clinical application of MSCs is limited by several hurdles, including senescence, low survivability in vivo, thrombosis, and tumorigenesis [8]. Furthermore, the direct use of MSCs in vivo is hindered by the trapping of intravascularly administered MSCs in the lungs or liver [9]. To overcome these limitations of using MSCs directly, extracellular vesicles (EVs) secreted by MSCs are recognized as efficient alternatives. EVs are nano-sized vesicles responsible for cell-to-cell communication in vivo, contributing to the maintenance of normal physiology and disease progression [10]. Furthermore, the therapeutic potential of stem cell-derived EVs in various preclinical models of degenerative diseases, including AKI, has been reported [8, 9, 11].

Despite the above advantages, obtaining a large number of EVs for clinical use remains a major hurdle, mostly because of the limited proliferation ability of MSCs [12]. Furthermore, MSCs are heterogeneous based on the isolation and culture protocols, donor characteristics, and batches, making standardization difficult [13]. Induced MSCs (iMSCs) are MSC-like cells that differentiate from induced pluripotent stem cells (iPSCs) and have various advantages over conventional MSCs. Most importantly, a large number of clonally derived iMSCs could be prepared, enabling the production of homologous EVs. Besides, quality control of parental cells and EVs is simple [14, 15]. The therapeutic potential of iMSC-derived EVs has been demonstrated in preclinical studies on inflammatory bowel disease, bone defects, periodontal disease, atherosclerosis, hepatic failure, and inflammation [16–21].

Recent studies have demonstrated that the biomolecular cargo of EVs can be modified by various methods, including cell priming or genetic modification [22, 23], depending on the nature of the pathological conditions [24, 25]. Among several options, preconditioning/priming strategies that use small molecules provide several advantages, as they are simple and fast, and the underlying mechanisms are well identified [22, 26]. Peroxisome proliferator-activated receptors (PPARs) are nuclear receptors comprising three isotypes, PPARα, PPARβ/δ, and PPARγ. PPARα and PPARβ/δ are well studied in hepatic cells, including hepatocytes, hepatic stellate cells, and Kupffer cells, and have been shown to participate in fatty acid catabolism and lipolysis [27, 28]. PPARγ is predominantly expressed in adipose tissue and prevents the excessive mobilization of fatty acids and adipokines from adipose tissues to other peripheral tissues, including the liver [29]. The efficacy of PPAR agonists has been investigated in treating metabolic diseases, including chronic liver injury and pulmonary hypertension, and cardiovascular diseases, including atherosclerosis [30]. In addition, it was recently shown that exosomes secreted from MSCs treated with rosiglitazone, a PPARγ agonist, accelerated diabetic wound healing by promoting collagen deposition and capillary formation compared with non-treated MSCs [31]. Lanifibranor, a pan-PPAR agonist, has anti-inflammatory and anti-fibrotic functions, as shown in clinical trials on type 2 diabetes and NAFLD [32, 33]. In tioacetamide (TAA)-induced cirrhotic rats, lanifibranor showed beneficial role in improving hepatic fibrosis by multiple mechanisms including enhancing liver microvascular function, decreased inflammation, and improving the integrity of live sinusoidal endothelial cells and hepatic stellate cells [33]. Our previous study demonstrated that EVs secreted from lanifibranor-stimulated iMSCs (pan PPAR-iMSC-EVs) potently reduced steatosis, inflammation, and ER stress in a mice model of non-alcoholic steatohepatitis (NASH). Also, pan PPAR-iMSC-EVs contributed to liver regrowth by inhibiting apoptosis and promoting proliferation [34].

In this study, we investigated the therapeutic potential of EVs obtained from iMSCs stimulated with or without a pan PPAR agonist in improving AKI. The present study reports the findings of comprehensive analyses in vitro using HK-2 cells and in vivo using a mouse model of cisplatin-induced AKI.

## Materials and methods

### Cell culture

HK-2 cells (Korean Cell Line Bank, Seoul, Korea) were cultured with RPMI 1640 (Welgene, Gyeongsan-si, Korea) supplemented with 10% FBS (Atlas Biologicals, Fort Collins, CO, USA) and 1% antibiotics-antimycotics (Genedirex, Taoyuan, Taiwan). The proliferation and survival of HK-2 was analyzed using a Cell Counting Kit-8 (CCK-8) (Dojindo Laboratories, Kumamoto, Japan) according to the manufacturer’s instructions. THP-1 monocytes (ATCC, Manassas, VA, USA) were cultured in RPMI 1640 (Welgene) supplemented with 10% FBS and 1% antibiotic–antimycotic solution. To differentiate THP-1 into M1 type, cells were stimulated with 200 ng/mL phorbol-12-myristate-13-acetate (PMA), 100 ng/ mL lipopolysaccharide (LPS), and 20 ng/mL IFNγ in RPMI 1640 medium supplemented with 10% FBS for 24 h. Subsequently, THP-1 monocytes were treated with 100 μg/mL of iMSC-EVs or pan PPAR-iMSC-EVs, 100 ng/mL LPS, and 20 ng/mL IFNγ in serum-free DMEM (Welgene, Gyeongsan-si, Korea). After 24 h, cells were subjected to RNA extraction and qPCR analysis.

### Culture of iMSCs

iMSCs were provided by Brexogen, Inc. (Seoul, Korea) and cultured as previously described [34]. Briefly, iMSCs were cultured in high-glucose DMEM (HyClone, Chicago, IL, USA) supplemented with 15% FBS and 1% antibiotic–antimycotic solution (Thermo Fisher Scientific, Waltham, MA, USA) at 37 °C in 5% CO_2_ and 95% humidified air. The cells were separated using TryPLE Express (Thermo Fisher Scientific) at 90% confluence and split into a 4-layer Cell Factory System (Thermo Fisher Scientific) at a density of 10,000 cells/cm^2^. Cells from passages 6–7 were used.

### Isolation of iMSC-EVs and pan PPAR-iMSC-EVs

DMEM (Gibco, Waltham, MA, USA) supplemented with 15% FBS was used for iMSC culture. EV-depleted FBS was prepared as previously described [35]. Cells from passages 6–7 were collected and seeded at a density of 10,000 cells/cm^2^ in a 150 mm culture dish (SPL, Pocheon-si, Korea). The next day, cells were treated with or without lanifibranor (Cayman, Ann Arbor, M, USA) for 24 h, after which the medium was replaced with DMEM supplemented with EV-depleted FBS and cultured for an additional 24 h. The supernatant was centrifuged for 10 min at 300×*g*, and the supernatant was transferred to a new tubes, which were then centrifuged for 20 min at 2000×*g*. This step was followed for another round of centrifugation at 10,000*g* for 80 min. The supernatant was passed through a 0.2-µm vacuum filter (Merck Millipore, Burlington, MA, USA). Finally, the EVs were isolated by ultracentrifugation at 100,000×*g* for 80 min, after which the pellet was washed with PBS again (Beckman Coulter, CA, USA). EV pellets were redissolved in EV-free PBS.

### RNA extraction and real-time qPCR

Total RNA was isolated from renal tissues and cells using TRIzol® (Ambion, Waltham, MA, USA), according to the manufacturer’s instructions. cDNA was synthesized from total RNA (1 μg) using a cDNA synthesis kit (PhileKorea Inc., Seoul, Korea). qPCR was conducted using the AccuPower® 2X GreenStar ® qPCR Master Mix (Bioneer, Daejeon, Korea) according to the manufacturer’s protocol. mRNA expression was analyzed using real-time qPCR (CFX96 Real-Time PCR System; Bio-Rad, Hercules, CA, USA). The primer sequences are listed in Additional file 1: Table S1. The expression of mRNA of the target genes relative to that of *Gapdh* was analyzed using the 2^−ΔΔCt^ method [36]. Each experiment was performed in triplicates.

### Animal experiments

All animal experiments were approved by the Institutional Animal Care and Use Committee of the Seoul National University (No. SNU-190413-6-1). BALB/c mice weighing 21–23 g were obtained from Koatech (Pyeongtaek, Korea) and maintained under specific pathogen-free conditions. Mice were randomly divided into several groups. Cisplatin (12 mg/kg) was intraperitoneally administered. At 8 and 24 h of cisplatin administration, 100 μL (400 μg) of iMSC-EVs or pan PPAR-iMSC-EVs were injected into the tail vein. All animals were euthanized by CO_2_ asphyxiation at 96 h after cisplatin injection.

### Fluorescence tracking of EVs

iMSC-ER or pan PPAR-iMSC-EVs were stained with 5 μM CellTracker™ Orange CMTMR tetramethylrhodamine (Thermo Fisher Scientific) for 30 min at 37 °C. The stained EVs were subsequently isolated by ultracentrifugation at 100,000×*g* for 80 min, after which the pellet was washed with PBS and ultracentrifuged again (Beckman Coulter). After redissolution in EV-free PBS, 100 μL (400 μg) of iMSC-EVs or pan PPAR-iMSC-EVs were injected into the tail vain 8 h after cisplatin administration. Mice were euthanized by CO_2_ asphyxiation 72 h after cisplatin injection. The kidneys were then embedded in an OCT compound (Sakura Finetek, Torrance, CA, USA) and sectioned at 10 μm using a cryotome (Leica, Wetzlar, Germany). Sections were then stained with phalloidin-iFluor 488 (Abcam, Cambridge, UK) according to the manufacturer’s instructions. Finally, the sections were mounted with a mounting medium containing DAPI (Vector Laboratories, Burlingame, CA, USA) and analyzed under a confocal microscope (Leica TCS SP8 STED; Leica Camera AG, Wetzlar, Germany).

### Serum biochemistry

Blood was collected via cardiac puncture and incubated at room temperature (23.0–25.2 °C) for 20 min in a BD Microtainer Serum Separator Tube (BD Biosciences). After centrifugation at 3000×*g* for 20 min, blood urea nitrogen and creatinine levels in the serum samples were measured using a Catalyst Dx Chemistry Analyzer system (IDEXX Laboratories, Inc., Westbrook, Maine).

### Injury scoring

Kidney tissues were fixed in 4% paraformaldehyde and processed for paraffin embedding. The sections were stained with hematoxylin and eosin for histological assessment. To quantify renal injury, the injury score was analyzed as previously described [37]. Briefly, the tubules were marked as viable (intact tubular morphology) or necrotic (totally disrupted tubules with loss of cuboidal cells) and counted in the stained tissue at 200× magnification. Tubules with thin cytoplasm containing fewer nuclei were considered injured. Tubules with more nuclei than normal cells were counted as recovered cells. Finally, the percentage (%) of each pattern of the total number of tubules was determined.

### Cryo-transmission electron microscopy

A 200-mesh copper grid (MiTeGen, Ithaca, NY, USA) coated with a formvar/carbon film was subjected to the hydrophilic treatment. The iMSC-EVs suspension (4 μL) was placed on a grid and blotted for 1.5 min at 100% humidity and 4 °C. iMSC-EVs on the grid were visualized at 36,000× magnification using a Talos L120C FEI transmission electron microscope (Thermo Fisher Scientific) at 120 kV.

### Nanoparticle tracking analysis

Measurements of the particle size distribution and concentration of iMSC-EVs and pan PPAR-iMSC-EVs were performed using a ZetaView Nanoparticle Tracking Analyzer PMX-120 instrument (Particle Metrix, Inning am Ammersee, Germany). Both types of EVs were diluted in sterile PBS to obtain the optimal volume for NTA. Measurements were performed at room temperature using a 488 nm laser and the high sensitive CMOS camera in several repeats. Sample analysis was conducted under the following camera settings and processing conditions: sensitivity 80, shutter 100, two cycles; 11 positions; NTA software version 8.05.14_SP7.

### Immunohistochemistry

Formalin-fixed, paraffin-embedded slides were deparaffinized in xylene and rehydrated in descending order of ethanol (100% to 70%). For antigen retrieval, Tris–EDTA (pH 9.0; Abcam; for CD31) or citrate buffer (pH 6.0; Abcam) was used according to the manufacturer’s instructions. The sections were incubated with primary antibodies (Additional file 1: Table S2) overnight at 4 °C. For chromogenic detection, an UltraVision LP Detection System HRP DAB kit (Thermo Fisher Scientific) was used according to the manufacturer’s instructions. After washing in PBST four times, reactivity was validated using a mouse/rabbit-specific HRP/DAB IHC Detection Kit (Micro-polymer, ab236466; Abcam). The slides were washed four times with distilled water and counterstained with Mayer's hematoxylin (4science, Gyeonggi-do, Korea) for 1.5 min at room temperature. After washing under running tap water, the slides were dehydrated in ascending order of ethanol (70%–100%). Images of representative fields were obtained using an Olympus BX43 light microscope (magnification, ×400; Olympus Corporation, Tokyo, Japan). Positively stained cells were quantified by calculating the mean number of positive cells from ten non-overlapping fields per slide at × magnification of ×400.

### Immunoblotting

Renal tissues were collected from the AKI mice and washed with ice-cold PBS. After tissues were lyzed with a RIPA buffer (Bio-solution, Suwon, Korea) containing a protease inhibitor cocktail (Roche, Basel, Switzerland) for 20 min, supernatant was collected after being centrifuged at 12,000×*g* for 15 min at 4 °C. The protein concentration was measured using a Pierce™ BCA Protein Assay Kit (Thermo Fisher Scientific). Protein extracts (20 μg) were separated by sodium dodecyl sulfate–polyacrylamide gel electrophoresis and transferred onto polyvinylidene difluoride membranes. The membrane was blocked with 5% nonfat dry milk or 5% BSA in Tris-buffered saline with 0.05% Tween-20 (TBST) and incubated overnight at 4 °C with primary antibodies. Detailed information on the primary antibodies used is provided in Additional file 1: Table S3. The membrane was then washed three times with TBST for 15 min each and incubated for 1 h at room temperature with corresponding horseradish peroxidase-conjugated secondary antibodies (Additional file 1: Table S3). The immunoreactivity of the antibodies was examined using an enhanced chemiluminescence kit (Thermo Fisher Scientific). Electrochemical images were obtained using an Azure imaging system (Azure Biosystems, CA, USA). Relative band densities were quantified using ImageJ software (version 1.50; National Institutes of Health, Bethesda, MD, USA).

### Statistical analysis

Statistical analyses were performed using analysis of variance, followed by Tukey’s multiple comparison test. All analyses were performed using the GraphPad Prism software (version 9.0; GraphPad, San Diego, CA, USA). Differences at *p* < 0.05 were considered significant.

## Results

### Characterization of iMSC-EV

**Fig. 1:**
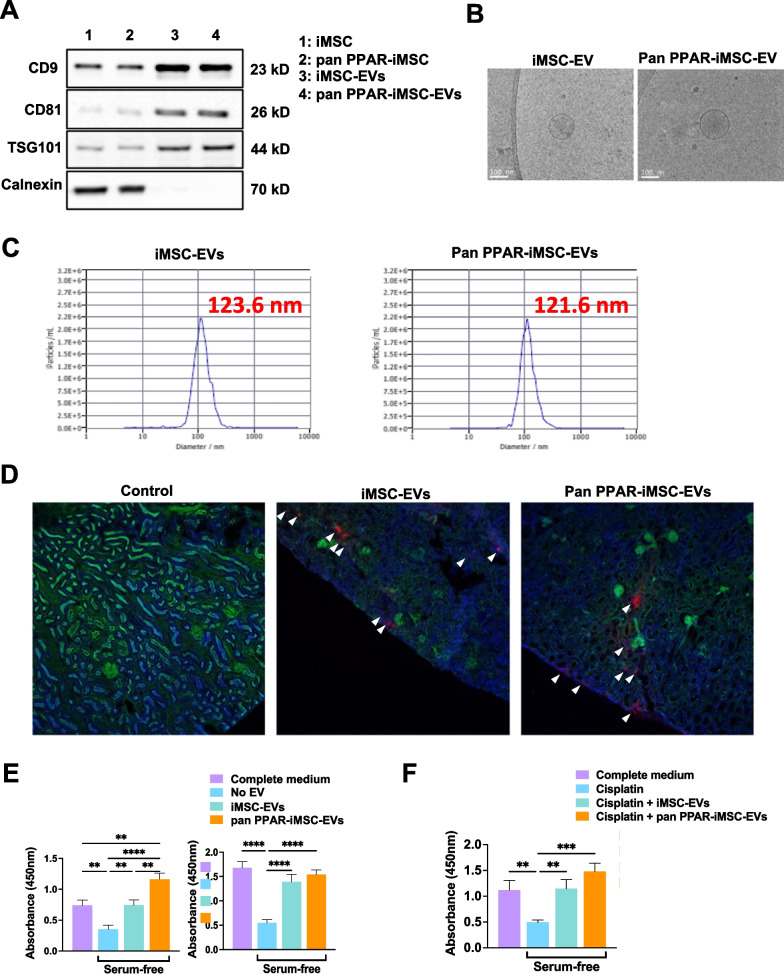
Characterization of iMSC-EVs and pan PPAR-iMSC-EVs. **A** Immunoblot detection of markers of extracellular vesicles in iMSC-EVs and pan PPAR-iMSC-EVs. The expression of markers for EVs (CD9, CD81, and TSG101) or organelles (calnexin) analyzed in iMSC-EVs and pan PPAR-iMSC-EVs. Full-length blots are presented in Additional file 2: Fig. S1. **B** The morphology of iMSC-EVs and pan PPAR-iMSC-EVs. Cryo-TEM was used for imaging. Scale bar: 100 nm. **C** Size distribution of iMSC-EVs and pan PPAR-iMSC-EVs measured using a nanotracking particle analyzer. **D** Detection of iMSC-EVs and pan PPAR-iMSC-EVs in the AKI kidney. iMSC-EVs or pan PPAR-iMSC-EVs were stained with CMTMR and subsequently intravascularly administered into mice 8 h after cisplatin treatment. After 72 h of cisplatin administration, the kidneys were harvested, and the presence of iMSC-EVs or pan PPAR-iMSCs was detected under laser confocal microscopy. **E** The effect of iMSC-EVs or pan PPAR-iMSC-EVs on the growth of HK2 cells. HK2 cells were cultured with iMSC-EVs or pan PPAR-iMSC-EVs under serum-free conditions for 24 (left) and 48 (right) h. The relative number of viable cells was determined by measuring the optical density (OD_450_) using a CCK-8 assay. **F** The effect of iMSC-EVs or pan PPAR-iMSC-EVs on the survival of HK2 cells undergoing cisplatin-mediated cell death. HK2 were treated with 15 μM of cisplatin for 24 h in the presence of iMSC-EVs or pan PPAR-iMSC-EVs. The relative number of viable cells was determined by measuring the optical density (OD_450_) using the CCK-8 assay

Immunoblot analysis revealed that the iMSC-EVs and pan-PPAR-iMSC-EVs were positive for EVs (CD9, CD91, and TSG101). These EV markers were also detected in the parental cells (iMSC and pan PPAR-iMSCs) (Fig. 1A; Additional file 2: Fig. S1). Calnexin, a cytoplasmic organelle protein, was only expressed in iMSC (Fig. 1A). The average size of the iMSC-EVs and pan-PPAR-iMSC-EVs was approximately 120 nm, as determined using cryo-TEM and NTA (Fig. 1B, C). To ascertain the incorporation of EVs into renal tissue, we intravascularly injected fluorescently labeled EVs into AKI mice and monitored their localization on day 3. As shown in Fig. 1D, iMSC-EVs and pan-PPAR-iMSC-EVs were detected in the renal tissues of AKI mice. We then explored whether pan-PPAR-iMSC-EVs have an enhanced potential for the growth of HK2. The results showed that both iMSC-EVs and pan-PPAR-iMSC-EVs could stimulate cell growth over a 48 h period, even without serum (*p* < 0.01 against vehicle). However, this effect was more significant in pan-PPAR-iMSCs than that in iMSC-EVs at 24 h (*p* < 0.01) (Fig. 1E). Next, we investigated whether these two EVs could reverse cisplatin-mediated cell death in HK2 cells. After 24 h of cisplatin treatment, the survival of HK2 was reduced to 40% compared to intact cells (*p* < 0.01). In contrast, the survival of HK2 cells treated with iMSC-EVs and pan PPAR-iMSC-EVs was restored to a similar degree as that of the positive control (HK2 cells cultured in complete medium). This effect was more significant in pan PPAR-iMSC-EVs (*p* < 0.001 vs. vehicle) than those in iMSC-EVs (*p* < 0.01 vs. vehicle) (Fig. 1F).

### Assessment of the therapeutic function of iMSC-EV AKI mice

**Fig. 2:**
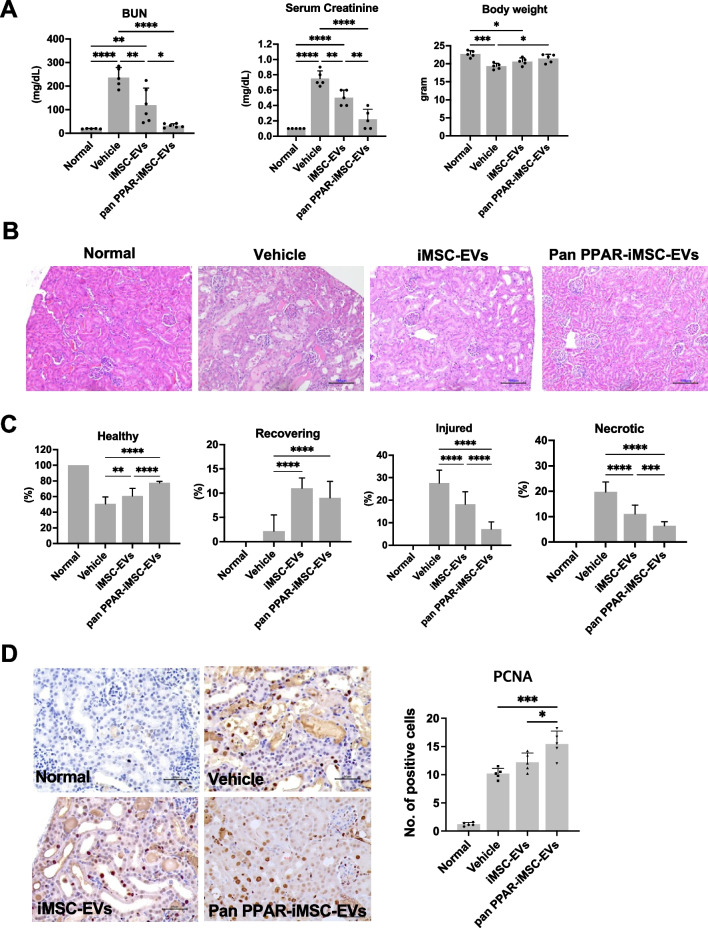
Assessment of therapeutic efficacy of EVs and pan PPAR-iMSC-EVs. iMSC-EVs or pan PPAR-iMSC-EVs were administered intravenously 8 and 24 h after cisplatin treatment. After 96 h of cisplatin treatment, mice were sacrificed. **A** The concentration of blood urea nitrogen, serum creatinine, and body weight. **B** Light microscopy images of kidney sections stained with hematoxylin and eosin. Magnification: 200x. **C** Assessment of renal injury (*N* = 4); Data are presented as mean ± sd. *p < 0.05; **p < 0.01; ***p < 0.001; ****p < 0.0001. **D** Immunohistochemical staining of proliferating cells with anti-PCNA antibody. Magnification: 400x. Scale bar: 50 μm. *p < 0.05; ***p < 0.001

We compared the renoprotective effects of iMSC-EVs and pan-PPAR-iMSC-EVs in a mouse model of cisplatin-induced AKI. Cisplatin treatment significantly reduced renal function, as shown by increased BUN and creatinine levels (*p* < 0.0001) and decreased body weight (*p* < 0.001) in treated mice compared to untreated mice. In contrast, both EV types markedly enhanced renal function; however, this effect was more significant in pan-PPAR-iMSC-EVs. In addition, body weight recovery was observed only in the pan-PPAR-iMSC-EV group (*p* < 0.05; Fig. 2A). Microscopic analysis of Hematoxylin & Eosin-stained tissue showed that AKI kidneys had a variety of tubular damage, including vacuolization, cell debris in the lumen, and hyaline casts. In contrast, this pathology was reduced by iMSC-EVs and was further ameliorated in the pan-PPAR-iMSC-EV-treated group (Fig. 2B). Next, we evaluated the degree of tubular damage in the kidneys by classifying them as healthy, recovering, injured, or necrotic based on a previous report [37]. Overall, both EVs reduced tubular damage compared to the vehicle, and pan-PPAR-iMSC-EVs showed an enhanced recovery of tubules over iMSC-EVs, as shown by healthier (*p* < 0.0001), less injured (*p* < 0.0001), and necrotic (*p* < 0.0001) tubules (Fig. 2C). To confirm recovery of the tubules, PCNA-reactive tubular cells were counted. More positive cells were found in AKI mice, and this increase was more significant in AKI mice that received pan-PPAR-iMSC-EVs than in those that received iMSC-EVs (*p* < 0.05) (Fig. 2D).

### Anti-inflammatory potential of pan PPAR-iMSC-EVs in AKI

**Fig. 3:**
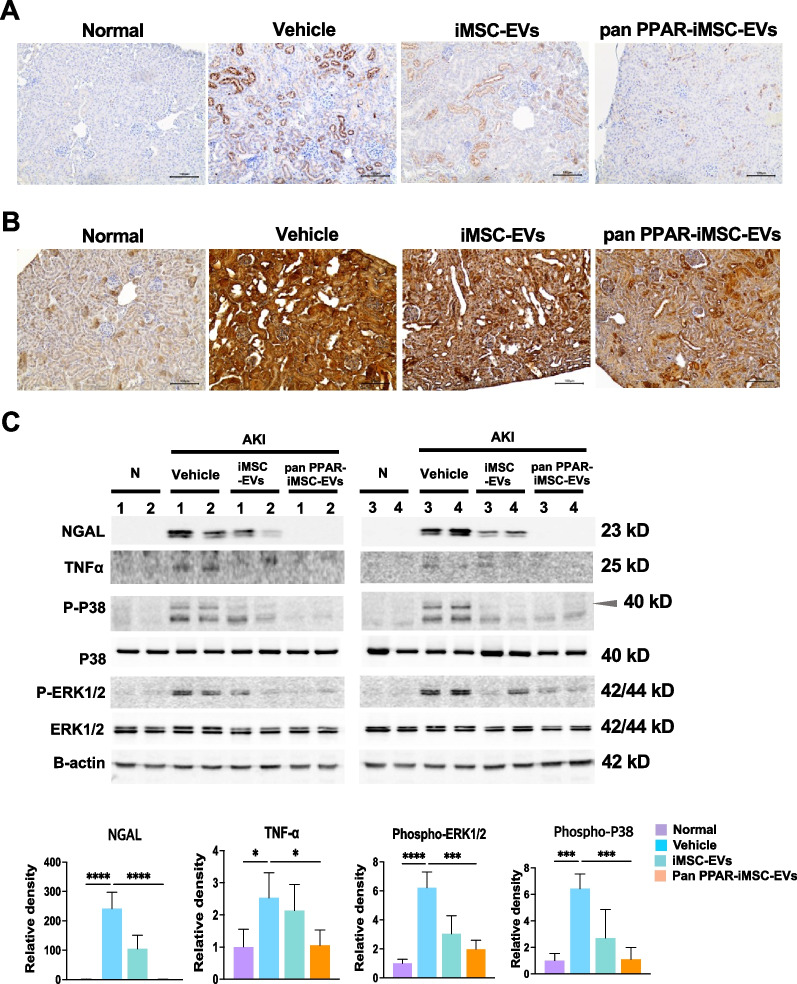
Alleviation of inflammation by pan PPAR-iMSC-EVs in AKI kidney. Immunohistochemical staining of NGAL (**A**) and TNF-α (**B**). (**C**) The protein expression of NGAL, TNF-α, phosphorylated P38 and ERK1/2 was compared among AKI animals that received the vehicle, iMSC-EV, or pan PPAR-iMSC-EVs. Each lane represents a sample from one animal. After antibodies corresponding to each protein were used for immunoblotting, the relative expression of the proteins was normalized against that of β-actin and quantified using ImageJ software; *N* = 4; Data are presented as mean ± sd. *p < 0.05; ***p < 0.001; ****p < 0.0001. *N* represents normal animals that had not undergone cisplatin-mediated AKI. Full-length blots are presented in Additional file 2: Figs. S2, S3

Tubular expression of neutrophil gelatinase-associated lipocalin (NGAL) increased in vehicle-treated AKI kidneys. However, its expression was reduced by iMSC-EVs; this reduction was greater in pan-PPAR-iMSC-EVs (Fig. 3A). Similarly, the expression of TNF-α was also induced in cisplatin-treated mice, whereas it reduced in iMSC-EV- and pan PPAR-iMSC-EV-treated mice, with the latter showing more pronounced reductions (Fig. 3B). Immunoblotting revealed that the protein expression of NGAL and TNF-α was augmented in AKI kidney. A slight reduction in both proteins was noted in iMSC-EV-treated animals compared to the vehicle-treated animals; however, the reduction was not significant. In contrast, in pan-PPAR-iMSC-EV-treated mice, the expression of both markers was significantly decreased compared to the vehicle group (NGAL, *p* < 0.0001; TNF-α, *p* < 0.05; Fig. 3C; Additional file 2: Figs. S2, S3). Phosphorylation of ERK1/2 and p38 was augmented in AKI mice, whereas a significant decrease in their activity was observed only in pan-PPAR-iMSC-EV-treated mice compared to those in vehicle-treated mice (phospho-ERK1/2, *p* < 0.001; phospho-p38, *p* < 0.001; Fig. 3C). qRT-PCR analysis revealed that mRNA expression of *Kim1, Il6, Timp1, Il1b, Il6Ra,* and *Ifngr2* was similarly decreased in iMSC-EV- and pan PPAR-iMSC-EV-treated mice compared to that in vehicle-treated AKI mice. Furthermore, the expression of several genes, including *Ngal, Ifngr1*, and *Atf4*, were significantly lower in pan-PPAR-iMSC-EV-treated mice than those in iMSC-EV-treated mice (*p* < 0.05, *p* < 0.01, and *p* < 0.05, respectively) (Additional file 2: Fig. S4A). In addition, pan-PPAR-iMSC-EVs significantly reduced the mRNA expression of inflammatory genes (*Mcp1, Tnfa, Cxcl10*, and *Il1b*) in activated THP-1 macrophages, whereas this reduction was less significant with iMSC-EVs (Additional file 2: Fig. S4B).

### Diminished immune cell deposition and improvement of capillary density by pan PPAR-iMSC-EVs

**Fig. 4:**
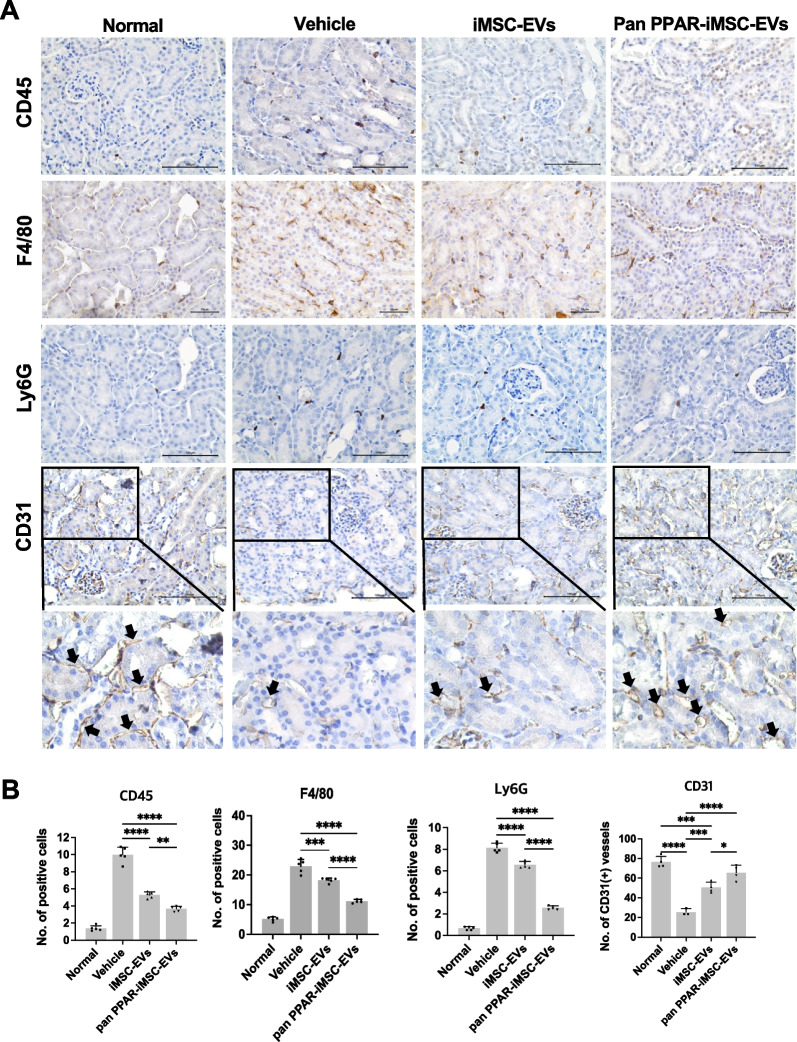
Immunohistochemical detection of inflammatory cells and measurement of capillary density in the AKI mice. **A** The expression of immune cell markers (CD45, F4/80, and Ly6G) and blood vessels (CD31) are shown; Scale bar: 100 μm. For CD31 staining, enlarged images of each figure are also provided. **B** Comparison of the number of infiltrated immune cells and capillary structures in AKI kidneys. Data are presented as mean ± sd; *p < 0.05; **p < 0.01; ***p < 0.001; ****p < 0.0001

Next, we examined the infiltration of various immune cells into the renal interstitium of AKI mice. More cells that reacted with antibodies against CD45 (pan-leukocytes), F4/80 (macrophages), and Ly6G (neutrophils) were found in the AKI kidneys (Fig. 4A, B). The number of cells expressing these markers was reduced by iMSC-EVs and pan-PPAR-iMSCs. However, this reduction was more significant in the pan-PPAR-iMSC-EV-treated mice than that in iMSC-EVs (CD45, *p* < 0.01; F4/80, *p* < 0.0001; Ly6G, *p* < 0.0001; Fig. 4A, B). The number of CD31-stained vessels was reduced in AKI mice, whereas capillary density was increased by iMSC (*p* < 0.001) and pan-PPAR-iMSC-EVs (*p* < 0.001). Notably, the recovery of capillary density was more significant in pan-PPAR-iMSC-EVs than that in iMSC-EVs (*p* < 0.05; Fig. 4A, B).

### Reduction of apoptosis by pan PPAR-iMSC-EVs

**Fig. 5:**
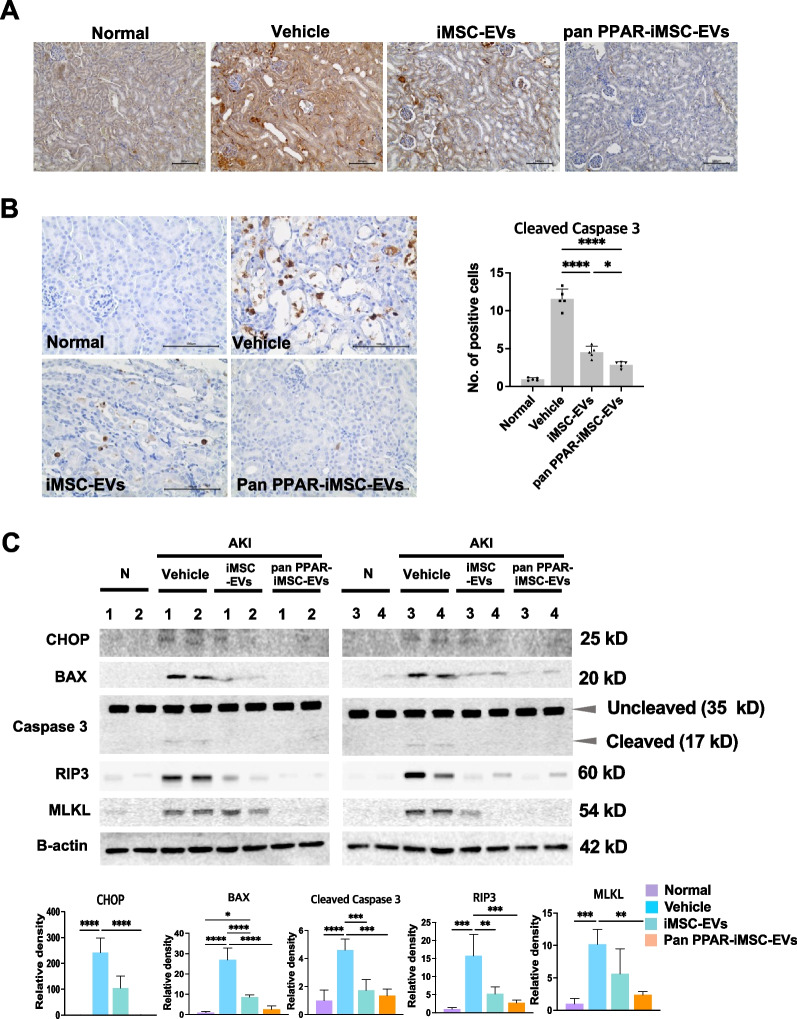
Effect of pan PPAR-iMSC-EVs on apoptotic injury in AKI. **A** Immunohistochemical detection of BAX protein expression in the kidney from AKI mice. Scale bars are 100 μm. **B** Detection of cleaved caspase-3-expressing cells in the kidney of AKI mice that received pan PPAR-iMSC-EVs; N = 4. Scale bar: 100 μm. Data are presented as mean ± sd. *p < 0.05; ****p < 0.0001. **C** Immunoblot analysis of apoptosis markers in AKI kidney. Single lane represents lysate from a single animal. Markers of ER stress (CHOP), apoptosis (BAX, caspase caspase 3) and necroptosis (RIP3, MLKL) were detected using corresponding antibodies. Their relative expression was normalized against that of β-actin and then quantified using ImageJ software; N = 4. Data are presented as mean ± sd. *p < 0.05; **p < 0.01; ***p < 0.001; ****p < 0.0001. Full-length blots are presented in Additional file 2: Fig. S5

Next, we explored whether pan PPAR-iMSC-EVs had a greater effect than iMSC-EVs on reducing apoptosis in AKI kidneys. As shown in Fig. 5A, AKI mice exhibited increased BCL2-Associated X (BAX) expression, wherein its expression was reduced in iMSC-EV- and pan-PPAR-iMSC-EV-treated mice, which was more pronounced in pan-PPAR-iMSC-EVs than that in iMSC-EVs (Fig. 5A, C). Furthermore, compared to those in the vehicle-treated mice, the number of cells that reacted against anti-cleaved caspase 3 antibody was increased in AKI mice, whereas it was reduced in iMSC-EV-treated (*p* < 0.0001) and pan PPAR-iMSC-EV-treated mice (*p* < 0.0001) (Fig. 5B). Moreover, this reduction was more significant in pan-PPAR-iMSC-EVs (*p* < 0.05). Next, we conducted immunoblot analysis to confirm the expression of apoptosis markers in AKI kidneys that received pan-PPAR-iMSC-EVs. The expression of markers for ER stress (CHOP), apoptosis (BAX and cleaved caspase 3), and necroptosis (RIP3 and MLKL) were augmented in AKI kidneys compared to normal mice (Fig. 5C; Additional file 2: Fig. S5). In contrast, the expression of these five markers was significantly reduced in animals that received pan-PPAR-iMSC-EVs compared to that in those received a vehicle (Fig. 5C). However, the downregulation of only BAX, cleaved caspase 3, and RIP3 was observed in MSC-EV-treated animals, indicating a less potent anti-apoptotic effect of MSC-EVs.

## Discussion

The pathogenesis of cisplatin-induced AKI is mostly attributable to the metabolic activation of cisplatin into a highly reactive substance that causes oxidative stress by disrupting the antioxidant system, producing inflammatory mediators (such as TNF-α, ICAM-1, and MCP1), leukocyte infiltration, endoplasmic reticulum stress, and apoptotic cell death [38, 39]. In the present study, we evaluated whether the therapeutic potential of EVs derived from iMSCs could be enhanced by pre-treating with a PPAR agonist. In serum-free cultures, pan-PPAR-iMSC-EVs were more effective in promoting HK-2 cell proliferation and survival during cisplatin-induced apoptosis. Mouse experiments demonstrated that renal function and tissue integrity were enhanced by both iMSC-EVs and pan-PPAR-iMSC-EVs, with the latter being more potent. Consistently, the proliferation of renal tubular cells was observed only in pan-PPAR-iMSC-EV-treated mice. Based on these observations, protein and mRNA expression analyses were conducted in AKI tissues to further confirm whether pan-PPAR-iMSC-EVs have enhanced renoprotective function compared to iMSC-EVs. qPCR, immunohistochemistry, and western blot analyses revealed that pan-PPAR-iMSC-EVs showed better therapeutic efficacy than iMSC-EVs against AKI.

Bruno et al. [41], for the first time, reported that microvesicles (MVs) from bone marrow-derived MSCs were able to inhibit the parameters of AKI induced by glycerol, which causes myolysis and hemolysis, leading to ischemic tubular injury [40]. However, this effect was abolished when EVs were treated with RNase, suggesting that at least a subset of RNA may contribute to AKI alleviation. Studies have also investigated the biological mechanisms of MSC-derived EVs. Zou et al. [42] demonstrated that MVs from Wharton’s jelly-derived MSCs (WJ-MSC-MVs) suppressed AKI induced by renal ischemia–reperfusion injury in rats, and WJ-MSC-MVs reduced apoptotic cell death within 48 h, with a reduction in the expression of CX3CL1, a chemo-attractant factor for macrophages found in endothelial cells. Furthermore, WJ-MSC-EVs reduced macrophage infiltration, improved renal function, and reduced fibrotic changes [41]. However, the studies demonstrating the function of iMSC-derived EVs in AKI are limited. Lim et al. reported that exosomes from iMSCs were able to attenuate the progression of AKI induced by unilateral ischemia and contralateral nephrectomy, as demonstrated by the enhanced renal microstructure, fewer apoptotic cells and oxidative DNA damage, increased expression of antioxidative and pro-inflammatory genes, and ERK1/2 activation [42]. However, the therapeutic effect of iMSC-derived exosomes was only demonstrated 24 h after ischemia; thus, it remains unknown whether their effect can be exerted at later phases of AKI (e.g., 48, 72, or 96 h).

The most common MSC priming strategy for increasing their anti-inflammatory function is temporarily exposing MSCs to an inflammatory milieu. For example, exosomes from IFN-γ-primed MSCs were superior at inhibiting Th17 differentiation than those from non-stimulated MSCs. Mechanistically, IFN-γ pretreatment led to an increase in miR-125a/125b in exosomes, which directly bind Stat3 and induced a subsequent reduction in Th17 differentiation [43]. Similarly, Harting et al. showed that the pretreatment of MSCs with TNF-α and IFN-γ yielded exosomes enriched in PGE2 and cyclooxygenase, which contributed to a marked decrease in TNF-α production by activated splenocytes [23]. Together with the anti-inflammatory function of pan PPAR-iMSC-EVs in NASH mice [34] as well as cisplatin-mediated AKI in the present study, these findings support that pan PPAR agonists may affect specific pathways in iMSCs, yielding distinct cargo biomolecules to reduce inflammation. Further studies are required to determine whether pan-PPAR-iMSC-EVs directly repress various immune cells.

Depending on the target disease or cell type, various priming strategies have been used to improve therapeutic outcomes in numerous models, including neurodegenerative and inflammatory diseases, myocardial infarction, colitis, osteoporosis, and liver fibrosis [44]. For example, neurotrophic factors (BDNF, HGF, and VEGF) have been used to prime MSCs in neurodegenerative diseases [45]. Another study showed that in hepatic fibrosis, decorin, a TGF-β inhibitor, was used for priming MSCs for hepatic fibrosis/cirrhosis [12]. In line with these priming strategies, it is ideal to use substances with well-established modes of action and low cytotoxicity [46]. Pan PPAR agonist has shown effective outcomes in clinical trials on type 2 diabetes, NAFLD, and cutaneous systemic sclerosis [32, 33]. In addition, our previous study demonstrated that pan-PPAR-iMSCs are enriched with gene signatures associated with cell proliferation (PI3K/AKT), regulation of inflammation, cell cycle, and apoptosis [34]. These findings are similar to those of the present AKI study, in which pan-PPAR-iMSC-EVs improved inflammation, ER stress, and tubular apoptosis/necrosis. In addition, this small molecule has already been used in clinical studies on metabolic diseases, indicating that it is safe, and our strategy is compatible with translational studies in the future [32].

Vascular injury is another potential therapeutic target for AKI. The potential of renal endothelium to regrow is low in contrast to that of tubular cells, and it was shown that vascular density could be reduced to 30–50% by renal ischemia–reperfusion injury [47]. Although the mechanism by which cisplatin causes endothelial loss is poorly understood, studies have shown that cisplatin induces the constriction of vascular smooth muscle cells (VSMCs) and a subsequent decrease in renal blood flow, leading to hypoxic damage. Furthermore, cisplatin treatment has been shown to increase oxidative stress, contributing to endothelial cell loss and compromised vascular relaxation via autoregulation. Consequently, the amount of NO derived from the endothelium may not be sufficient because of reduced endothelial cells or eNOS function, which can lead to sustained vascular constriction and subsequent hypoxia [5]. Few studies have examined whether EVs can recover from capillary rarefaction in renal disease models. In a renal ischemia–reperfusion mouse model, it was demonstrated that EVs isolated from mouse renal MSCs were capable of increasing renal capillary density, as shown by increased CD31-positive cells and PCNA-positive cells and reduced TUNEL( +) cells in peritubular capillaries [48]. Additionally, the aforementioned roles of EVs were similar to those of the parental renal MSCs, indicating that recovery from ischemia-induced vascular injury is mostly attributable to EVs. Another study showed that renal MSC-derived EVs were functional in re-equipped mice with renal capillary loss in tubulointerstitial fibrosis induced by a unilateral ureteral obstruction in mice [49].

It should be noted that the exosome delivery method used in this study may not be optimal. Intravascular administration of exosomes in vivo has limitations, such as a short duration due to loss of integrity or instantaneous degradation and localization into non-target organs (e.g., lungs or liver). Several attempts have been made to increase the bioavailability and localization of exosomes. For example, inserting exosome-loaded hydrogels underneath the renal capsule after injury increases the entry of exosomes into kidney tissues, especially in the cortical area [50]. Exosome patches engineered using tissue scaffolds are ideal options [51]. Another alternative is to use cell sheets engineered with MSCs, where exosomes might be secreted and transferred to the lesions for an extended period. A recent study demonstrated that in a rat model of renal fibrosis induced by ischemia/reperfusion, a bone marrow-derived MSC (BMMSC)-engineered cell sheet attached to the kidney was able to inhibit renal fibrosis by increasing the microvascular density in rats; however, the secretion of EVs was not demonstrated [52]. Another breakthrough that can overcome organ specificity is the optimization of the delivery route. Ullah et al. [54] showed the potential for the intra-arterial delivery of exosomes, which enhanced renal function, proliferation, tissue regeneration, and reduced inflammation, contributing to the inhibition of cisplatin-induced AKI [53].

Although pan-PPAR-iMSC-EVs have shown enhanced protective function against AKI compared with iMSC-EVs, many issues remain unsolved. Most importantly, the exact molecular mechanisms by which these drugs block AKI need to be determined. Based on the results of our previous study [34], several candidate pathways, including those associated with inflammatory responses (i.e., chemokine signaling, NF-κB, PPAR signaling), may have contributed to an enhanced renoprotection by pan PPAR-iMSC-EVs [34]. Similarly, the present study suggest that lanifibranor stimulation of iMSCs can increase the anti-inflammatory role of EVs, as evidence by the reduced expression of various inflammatory chemokines/cytokines as well as infiltration of innate immune cells (Fig. 4 and Fig. S4). Furthermore, other biomolecules, such as DNA and RNA in EVs could also have influenced the physiological responses of target cells. Therefore, identifying key biomolecules (e.g., RNAs or proteins) critical for promoting growth or suppressing apoptosis of tubular cells by loss-of-function or rescue studies is needed to better characterize the renoprotective roles of pan PPAR-iMSC-EVs.

## Conclusion

The results of the present study indicated that EVs secreted from pan-PPAR agonist-treated iMSCs have a greater renoprotective effect against AKI. Pan PPAR-iMSC-EVs reduced AKI by playing multiple renoprotective roles, including inhibiting apoptosis and inflammation and promoting tubular cell regrowth and renal capillaries. Our strategy represents an alternative therapeutic option for the treatment of AKI.

### Supplementary Information

**Additional file 1:Table S1** Primer sequences used for qRT-PCR. **Table S2** Information on the antibodies used for immunohistochemical staining. **Table S3** Information on the antibodies used for immunoblotting.

**Additional file 2: Figures**: **Fig. S1** Full-length blots for EV immunoblotting. **Fig. S2** Full-length blots for inflammatory markers. **Fig. S3** Full-length blots for β-actin used for inflammatory markers. **Fig. S4** qPCR analysis in AKI kidney tissues and THP-1 cells. **Fig. S5** Full-length blots for apoptosis markers and β-actin.

**Additional file 3: Figure Legends**.

## Data Availability

The data and materials can be provided upon request via email to the corresponding author.

## Abbreviations

AKI: Acute kidney injury
BAX: BCL2-associated X
BDNF: Brain-derived neurotrophic factor
BMMSC: Bone marrow-derived mesenchymal stem cell
CCK-8: Cell counting kit-8
CKD: Chronic kidney disease
CHOP: CCAAT-enhancer-binding protein homologous protein
CXCL1: C-X-C motif chemokine ligand 1
DPP4: Dipeptidyl peptidase 4
ERK1/2: Extracellular signal‑regulated protein kinase ½
HGF: Hepatocyte growth factor
HK-2: Human kidney 2
ICAM-1: Intercellular adhesion molecule-1
IFN-γ: Interferon-gamma
MCP-1: Monocyte chemoattractant protein-1
MLKL: Mixed lineage kinase domain-like pseudokinase
MSC: Mesenchymal stem cell
MV: Microvesicle
NAFLD: Non-alcoholic fatty liver disease
NF-κB: Nuclear factor kappa-light-chain-enhancer of activated B cells
NGAL: Neurtrophil gelatinase-associated lipocalin
PBS: Phosphate buffered-saline
PCNA: Proliferating cell nuclear antigen
PPAR: Peroxisome Proliferator-Activated Receptor
RIPA: Radioimmunoprecipitation assay
RIP3: Receptor-interacting protein kinase 3
STAT3: Signal transducer and activator of transcription 3
TEM: Transmission electron microscopy
TGF-β: Transforming growth factor-β
TNF-α: Tissue necrosis factor-α
TUNEL: TdT-mediated dUTP-biotin Nick End Labeling)
VEGF: Vascular endothelial growth factor
VSMC: Vascular smooth muscle cell
