# Supplementary material for: Pan PPAR agonist stimulation of induced MSCs produces extracellular vesicles with enhanced renoprotective effect for acute kidney injury
Source: Stem Cell Res Ther. 2024 Jan 2;15:9. doi: 10.1186/s13287-023-03577-0 (PMC10763307; doi:10.1186/s13287-023-03577-0)
Supplement: Supplementary file 1 — Additional file 1:Table S1 Primer sequences used for qRT-PCR. Table S2 Information on the antibodies used for immunohistochemical staining. Table S3 Information on the antibodies used for immunoblotting. [file 13287_2023_3577_MOESM1_ESM.docx]

Supplementary Table S1. Primer sequences used for qRT-PCR.

| Genes | Forward (5’>3’) | Reverse (5’>3’) |
| --- | --- | --- |
| Mouse NGAL | GCAGGTGGTACGTTGTGGG | CTCTTGTAGCTCATAGATGGTGC |
| Mouse IL-6Rα | CCTGAGACTCAAGCAGAAATGG | AGAAGGAAGGTCGGCTTCAGT |
| Mouse KIM-1 | TCCACACATGTACCAACATCAA | GTCACAGTGCCATTCCAGTC |
| Mouse TIMP-1 | CGAGACCACCTTATACCAGCG | ATGACTGGGGTGTAGGCGTA |
| Mouse IL-6 | CTGCAAGAGACTTCCATCCAG | AGTGGTATAGACAGGTCTGTTGG |
| Mouse IL-1β | GAAATGCCACCTTTTGACAGTG | TGGATGCTCTCATCAGGACAG |
| Mouse IL-8 | ATGGCTGCTCAAGGCTGGTC | AGGCTTTTCATGCTCAACACTAT |
| Mouse MCP-1 | AGGTCCCTGTCATGCTTCTG | TCTGGACCCATTCCTTCTTG |
| Mouse IFNγR1 | CTGGCAGGATGATTCTGCTGG | GCATACGACAGGGTTCAAGTTAT |
| Mouse IFNγR2 | TCCTCGCCAGACTCGTTTTC | GTCTTGGGTCATTGCTGGAAG |
| Mouse ATF4 | GTTCTCCAGCGACAAGGCTA | ATCCTGCTTGCTGTTGTTGG |
| Mouse GAPDH | TGGCAAAGTGGAGATTGTTGCC | AAGATGGTGATGGGCTTCCCG |
| Human MCP-1 | GACCATTGTGGCCAAGGAGAT | TGTCCAGGTGGTCCATGGA |
| Human TNF-α | GAGCTGAACAATAGGCTGTTCCCA | AGAGGCTCAGCAATGAGTGACAGT |
| Human CXCL10 | TGGCATTCAAGGAGTACCTCTC | TGATGGCCTTCGATTCTGGA |
| Human IL-1β | ACAGCTGGAGAGTGTAGATCC | CTTGAGAGGTGCTGATGTACC |
| Human GAPDH | GAGTCAACGGATTTGGTCGT | TTGATTTTGGAGGGATCTCG |

Supplementary Table S2. Information on the antibodies used for immunohistochemical staining

| Target | Primary antibodies | Secondary antibodies and chromogenic kit |
| --- | --- | --- |
| PCNA | Mouse mAb against PCNA, Santa Cruz Biotechnology, sc-56, 1:100 | Mouse and Rabbit Specific HRP/DAB IHC Detection Kit - Micro-polymer (ab236466), Abcam |
| NGAL | Rabbit mAb against NGAL, Abcam, ab216462, 1:2,000 |  |
| CD45 | Rabbit pAb against CD45, abcam, ab10558, 1μg/ml |  |
| F4/80 | Rabbit mAb, Cell Signaling Technology, #70076T, 1:250 |  |
| Ly6G | Anti-Ly6g antibody (EPR22909-135), abcam, ab238132, 1:600 |  |
| TNF-α | Anti-TNF alpha antibody (52B83), abcam, ab1793,1:50 |  |
| Cleaved caspase 3 | Cleaved Caspase-3 (Asp175) Antibody, Cell Signaling Technology, 9661, 1:400 |  |
| CD31 | Anti-CD31 antibody (RM1006), abcam, ab281583, 1:4,000 |  |
| BAX | Bax Antibody (B-9), Santa Cruz Biotechnology, sc-7480, 1:100 |  |

Supplementary Table S3. Information on the antibodies used for immunoblotting

| Target | Primary antibodies and dilution ratio | Secondary antibodies |
| --- | --- | --- |
| NGAL | Rabbit mAb against NGAL, Abcam, ab216462, 1:1,000 | Goat anti-Rabbit IgG H&L (HRP), ab6721, Abcam, 1:10,000  Rabbit anti-Mouse IgG H&L (HRP), ab6728, Abcam, 1:10,000  Goat anti-Rat IgG H&L (HRP), ab97057, Abcam, 1:10,000 |
| TNF-α | Mouse mAb against TNF-α, Abcam, ab1793, 1:1,000 |  |
| Phospho-ERK1/2 | Rabbit mAb against p44/42 (ERK1/2) (Thr202/Tyr204) (D13.14.4E) XP, Cell Signaling Technology, #4370, 1:1,000 |  |
| ERK1/2 | Rabbit mAb against p44/42 (ERK1/2) (137F5), Cell Signaling Technology, #4695, 1:1000 |  |
| Caspase 3 | Rabbit pAb against caspase-3, Cell Signaling Technology, #9662, 1:400 |  |
| CHOP | Mouse mAb against CHOP (L63F7), Cell Signaling Technology, #2895, 1:1,000 |  |
| Phospho-P38 | Rabbit mAb against phospho-p38 MAPK, Cell Signaling Technology, #4511, 1:1,000 |  |
| P38 | Rabbit mAb against p38, Cell Signaling Technology, #8690, 1:1,000 |  |
| BAX | Rabbit pAb against BAX, Cell Signaling Technology, #2772, 1:1,000 |  |
| RIP3 | Rabbit mAb against RIP3, Cell Signaling Technology, #95702, 1:1000 |  |
| MLKL | Rat mAb against MLKL, abcam, #243142, 1:1000 |  |
| B-actin | Mouse mAb against β-actin, sc-47778, 1:1,000 |  |
