# Supplementary figures and images for: Pan PPAR agonist stimulation of induced MSCs produces extracellular vesicles with enhanced renoprotective effect for acute kidney injury
Source: Stem Cell Res Ther. 2024 Jan 2;15:9. doi: 10.1186/s13287-023-03577-0 (PMC10763307; doi:10.1186/s13287-023-03577-0)

**Fig S1**

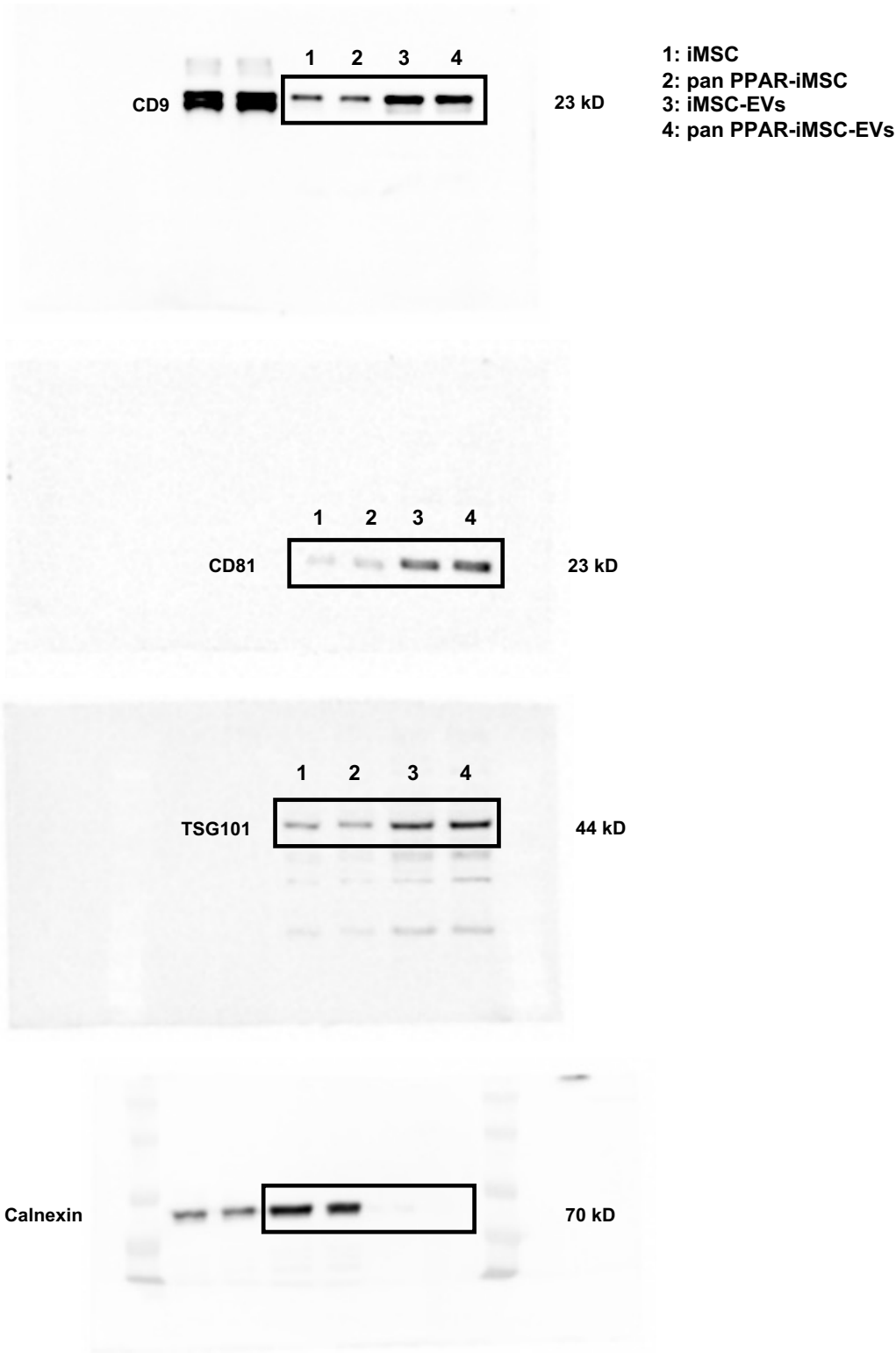

Fig S2

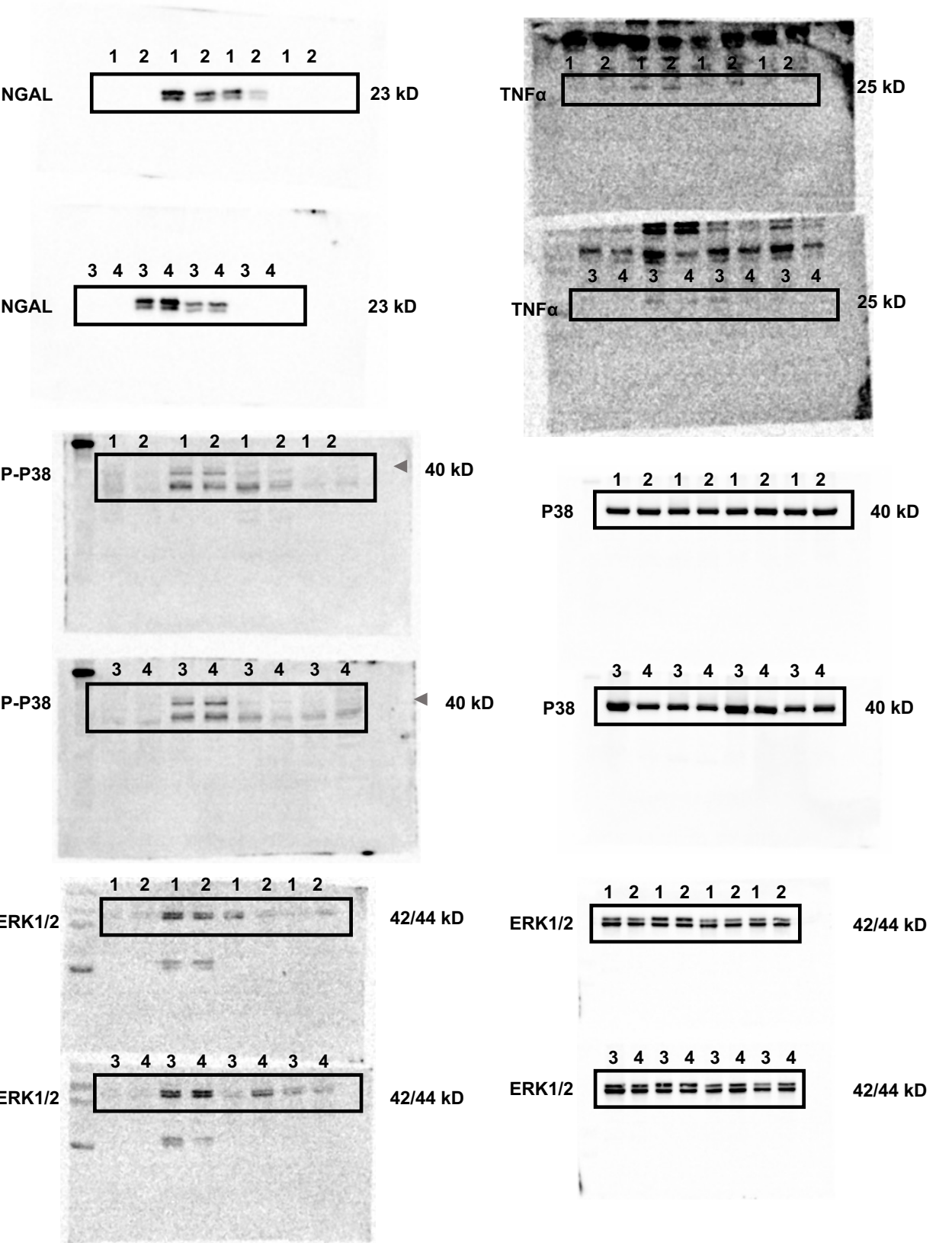

**Fig S3**

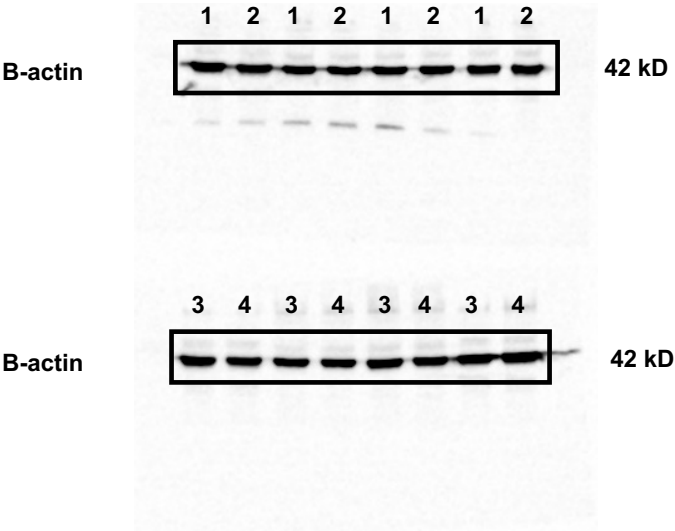

**Fig S4**

**A**

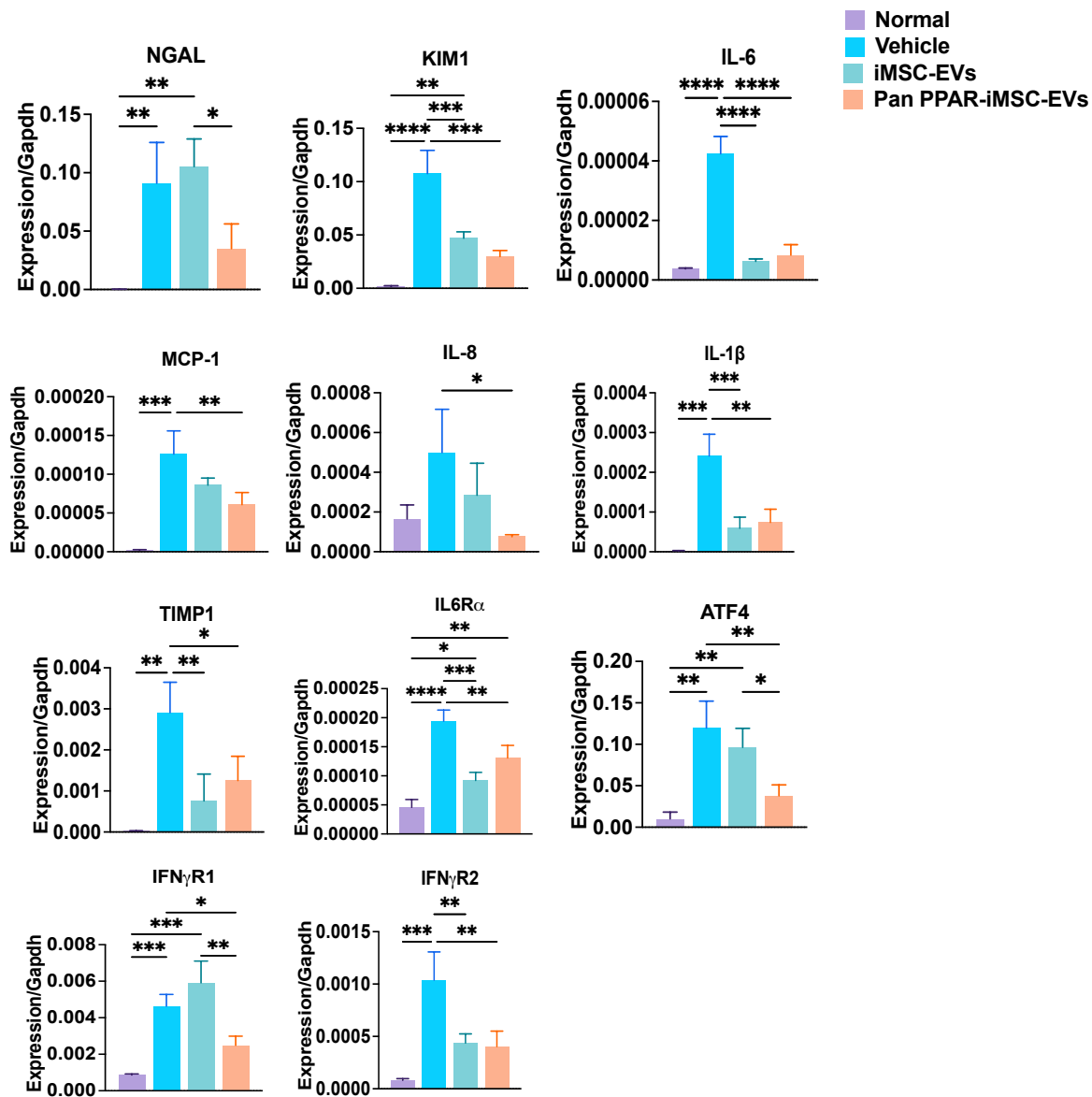

**B**

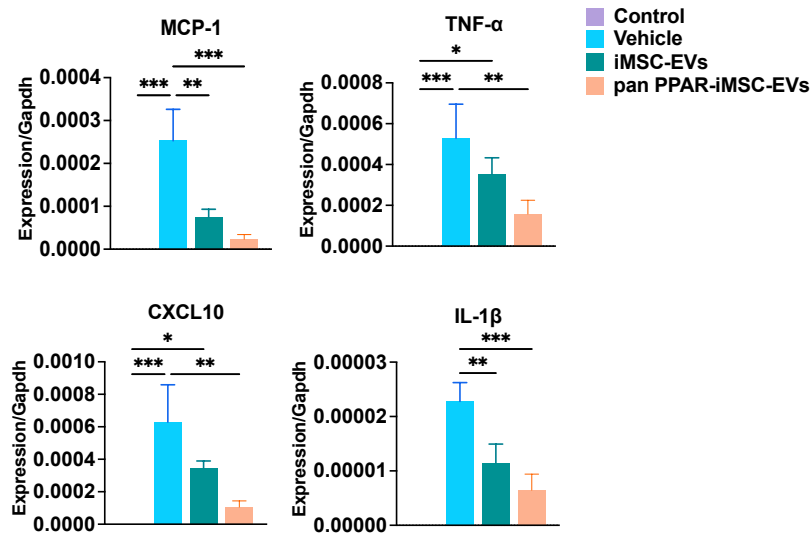

Fig S5

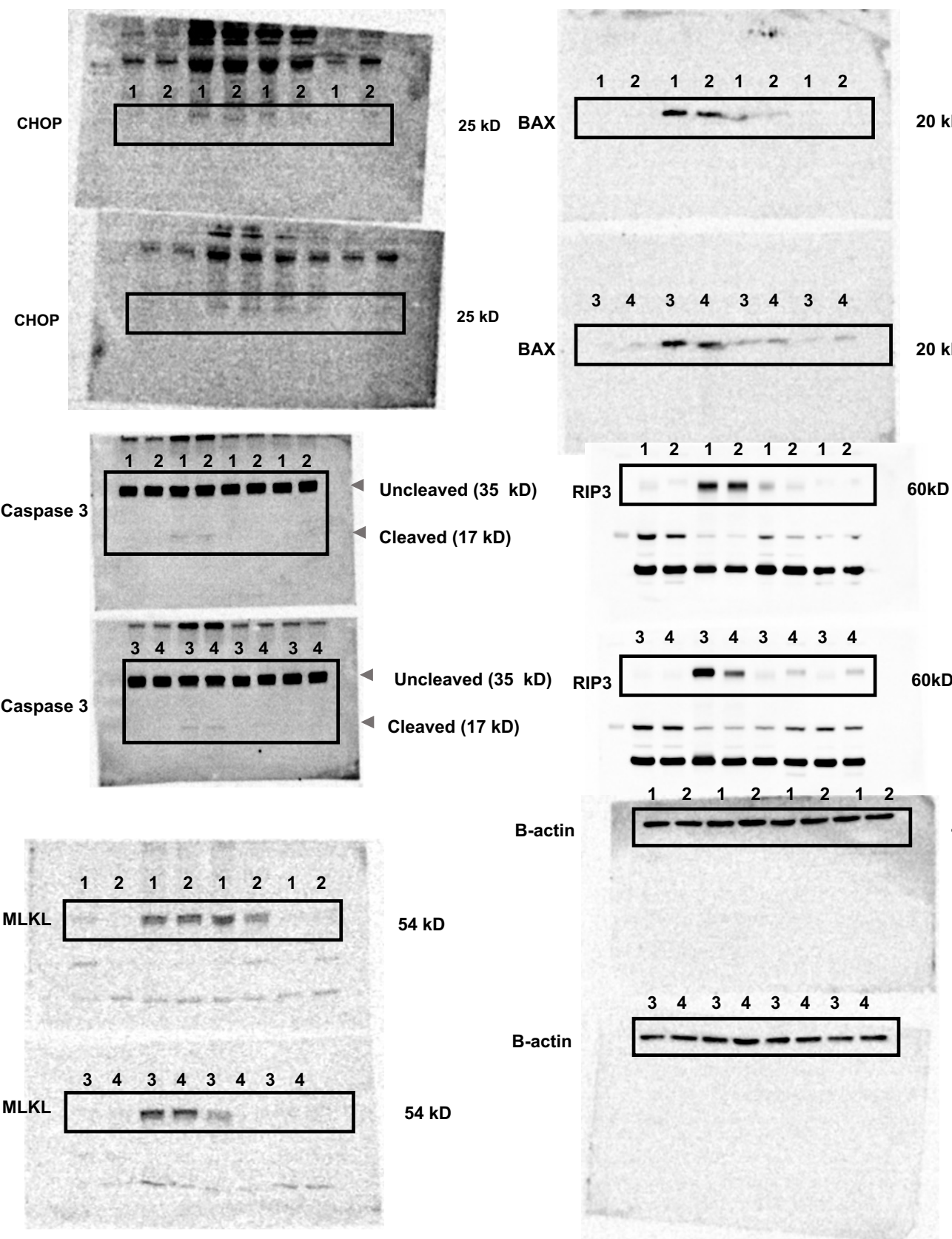

Supplement: Supplementary file 2 — Additional file 2: Figures: Fig. S1 Full-length blots for EV immunoblotting. Fig. S2 Full-length blots for inflammatory markers. Fig. S3 Full-length blots for β-actin used for inflammatory markers. Fig. S4 qPCR analysis in AKI kidney tissues and THP-1 cells. Fig. S5 Full-length blots for apoptosis markers and β-actin. [file 13287_2023_3577_MOESM2_ESM.pdf]
