# Supplementary material for: Pan PPAR agonist stimulation of induced MSCs produces extracellular vesicles with enhanced renoprotective effect for acute kidney injury
Source: Stem Cell Res Ther. 2024 Jan 2;15:9. doi: 10.1186/s13287-023-03577-0 (PMC10763307; doi:10.1186/s13287-023-03577-0)
Supplement: Supplementary file 3 — Additional file 3: Figure Legends. [file 13287_2023_3577_MOESM3_ESM.docx]

**Fig. S1** Full-length blots for EV immunoblotting.

**Fig. S2** Full-length blots for inflammatory markers. Numbers in each lane indicate protein samples from a single animal.

**Fig. S3** Full-length blots for β-actin used for inflammatory markers. Numbers in each lane indicate protein samples from a single animal.

**Fig. S4** qRT-PCR analysis in AKI kidney tissues and THP-1 macrophages. (A) qRT-PCR analysis of inflammatory genes in the kidneys. The relative expression of each gene was normalized against that of *Gapdh*; N = 4. Data are presented as mean ± sd. **p* < 0.05; ***p* < 0.01; ****p* < 0.001; *****p* < 0.0001. (B) qRT-PCR analysis of inflammatory genes in LPS/IFNγ‐stimulated THP‐1 macrophage. The relative expression of each gene was normalized against that of *Gapdh*. N = 4; Data are presented as mean ± sd; *p < 0.05; **p < 0.01; ***p< 0.001

**Fig. S5** Full-length blots for apoptosis markers and β-actin. Numbers in each lane indicate protein samples from a single animal.
